# One-Step Nucleic Acid Amplification System in Comparison to the Intraoperative Frozen Section and Definitive Histological Examination Among Breast Cancer Patients: A Retrospective Survival Study

**DOI:** 10.3389/fonc.2022.847858

**Published:** 2022-05-18

**Authors:** Serena Bertozzi, Ambrogio P. Londero, Michela Bulfoni, Luca Seriau, Diane Agakiza, Alberto Pasqualucci, Michela Andretta, Maria Orsaria, Laura Mariuzzi, Carla Cedolini

**Affiliations:** ^1^ Breast Unit, University Hospital of Udine, Udine, Italy; ^2^ Ennergi Research, Lestizza, Italy; ^3^ Department of Medical Area (DAME), University of Udine, Udine, Italy; ^4^ Academic Unit of Obstetrics and Gynaecology, Department of Neuroscience, Rehabilitation, Ophthalmology, Genetics, Maternal and Infant Health, University of Genoa, Genova, Italy; ^5^ Institute of pathology, University Hospital of Udine, Udine, Italy; ^6^ Department of Surgical and Biomedical Science, University of Perugia, Perugia, Italy; ^7^ Rashid Hospital, Trauma and Emergency Center, Dubai Health Authority, Dubai, United Arab Emirates

**Keywords:** Keywords sentinel lymph node biopsy, OSNA, breast cancer, survival, frozen section

## Abstract

**Simple Summary:**

Implementing intraoperative assessment of sentinel lymph nodes by one-step nucleic acid amplification in early breast cancer can reduce the surgical burden to the patient and the costs to the health system. However, only limited data are available in terms of long-term disease-free survival and overall survival. Therefore, this study aims to compare disease-free survival and overall survival between one-step nucleic acid amplification, frozen section, and definitive histology. These results could impact the healthcare community, adding further proof to the body of evidence supporting the broader adoption of this innovative technology that enables a safe reduction in patient surgical burden and healthcare costs.

**Background:**

The one-step nucleic acid amplification (OSNA) system is a novel molecular technique, which consents to quick intraoperative detection of sentinel lymph node metastases by the amplification of cytokeratin 19 mRNA. Our study aims to evaluate the OSNA method in comparison with frozen section (FS) and definitive histological examination of the sentinel lymph node biopsy among early breast cancer patients considering disease-free survival (DFS) and overall survival (OS).

**Methods:**

In this study, we included all women who underwent sentinel lymph node biopsy (SLNB) for breast cancers classified as TNM stage I and II in our center between January 2005 and January 2017, and the follow-up was collected up to January 2019. We divided patients among three groups based on SLNB evaluation: definitive histological examination, intra-operative FS, or OSNA.

**Results:**

We included 2412 SLNBs: 727 by definitive histological examination, 697 by FS, and 988 by OSNA. Isolated tumor cells were found in 2.32% of cases, micrometastasis in 9.12%, and macrometastases in 13.64%. Surgical procedure duration was significantly shorter in OSNA than in FS (42.1 minutes ±5.1 vs. 70.1 minutes ±10.5, p <0.05). No significant differences have been observed among the three groups regarding OS, DSF, cumulative local, or distant metastases. In particular 5-year DFS was 96.38% in definitive histology (95% C.I. 95.02-97.75%), 96.37% in FS (95% C.I. 94.98-97.78%), and 96.51% in OSNA group (95% C.I. 95.32-97.72%).

**Conclusions:**

No difference in OS and DFS was found comparing OSNA, FS, and definitive histology. Furthermore, reduced operative time was found in the OSNA group.

## Introduction

The sentinel lymph node biopsy (SLNB) procedure has dramatically revolutionized breast surgery during the last decades (1). In fact, SLNB with staging intent has progressively replaced complete axillary lymph node dissection (CALND), previously intended with a curative purpose. Probably in the future, even this procedure might be abandoned in favor of a non-surgical lymph node evaluation to predict patients’ prognosis and better tailor subsequent therapies (1, 2).

For what concerns the technique, we assisted in a first evolution to reduce the number of interventions with the introduction of intraoperative frozen section evaluation of the SLNB. Performing in the same surgical session, the primary breast surgery, the SLNB, and eventually the CALND according to the intraoperative lymph node assessment reduces the patients’ surgical burden and the healthcare system costs (3–5). Secondly, an intraoperative molecular-based lymph node staging has been adopted in place of the traditional morphological examination to minimize the operative time and enhance accuracy (6). In particular, the one-step nucleic acid amplification (OSNA) system consists of the amplification of cytokeratin (CK) 19 mRNA directly from the lysate to distinguish positive from negative samples (7–9). This second advance, besides ensuring a reduction in surgical sessions per patient and the costs for the healthcare system, allows reducing operating times and the pathologist workload (4, 6).

Although OSNA is considered the most accurate intraoperative lymph node staging technique (10), the literature lacks cohort studies, with everyday routine data, comparing survival analysis between OSNA and other lymph node staging methods (intraoperative frozen section or definitive histology).

Our study evaluates the OSNA method for the intraoperative analysis of sentinel lymph node biopsy compared with frozen section and definitive histological examination among patients affected by breast cancers classified as TNM stage I and II considering disease-free survival and overall survival.

## Methods

### Study Design and Subjects

All women were included in this retrospective cohort study who underwent SLNB for invasive breast cancers classified as TNM stage I and II in our center between January 2005 and January 2017. The follow-up was collected up to January 2019. According to Helsinki Declaration, the study was carried out and followed the dictates of the general authorization to process personal data for scientific research purposes by the Italian Data Protection Authority. We excluded all cases that underwent primary CALND, male breast cancer patients, women affected by intraductal neoplasia, benign breast diseases, as well as invasive breast cancers classified as TNM stage III or IV. The patient information was gathered from clinical files.

In all included cases, SLNB was performed. At the same time, breast cancer removal consisted of breast-conserving surgery or mastectomy when appropriate, followed or not by immediate breast reconstruction as previously described (5, 11, 12). Non-palpable breast lesions were removed by radio-guided occult lesion localization or wire hook localization as previously described (5, 13–15).

The cohort of included patients was divided into three groups according to SLNB histological assessment: group A consists of all cases in which SLNB was assessed by definitive histological examination, group B includes all cases in which SLNB was assessed by intraoperative frozen section (FS), and group C includes all cases in which SLNB was assessed by OSNA. Intraoperative FS was introduced in 2002 and is still performed in selected cases (more than three sentinel nodes, big-sized sentinel nodes, history of hematological disease, previous neoadjuvant chemotherapy, OSNA system unavailability). OSNA system was introduced in October 2011. Definitive histological examination was performed on any sentinel node removal under local anesthesia before planning final breast surgery.

### Definitive Histological Examination

In the event of definitive histological examination, all biopsied lymph nodes were cut in parts of 2 mm thickness, formalin-fixed, and paraffin-embedded before undergoing an accurate in toto evaluation of 0.15-mm-spaced, hematoxylin-eosin-stained sections (16). Concurrently, an immunohistochemical assessment of a random portion of the considered nodes to search for an eventual positivity for cytokeratins was performed on pathologist request (17).

### Intraoperative Frozen Section

In intraoperative FS, the sentinel nodes were cut in parts of 2 mm thickness, frozen, and optimal cutting temperature (OCT) embedded before undergoing intraoperative assessment. First, the pathologist performed a histological examination of 2 hematoxylin-eosin-stained sections (0.15-mm-spaced). Thereafter, the remnant sentinel lymph node tissue underwent traditional definitive in toto histological examination with evaluation of 0.15-mm-spaced, hematoxylin-eosin-stained sections, and immunohistochemical evaluation of a random nodal portion on pathologist request.

### One-Step Nucleic Acid Amplification

The detailed OSNA assay has been previously described (18–20). First, all the collected sentinel lymph nodes were separately homogenized in an mRNA-stabilizing solution (Lynorhag, pH 3.5 Sysmex^®^). Then, an isothermal (65°C) CK19 amplification was performed using the Lynoamp amplification kit (Sysmex^®^) through a reverse transcriptase amplification assay (RT-LAMP) in a gene amplification detector RD-100i (Sysmex^®^). A standard positive control sample and a negative control sample were used for calibration in every assay. Our protocol complied with a previously described procedure (20). As previously defined, the results were given automatically in a semiquantitative way (18, 20–22). In brief, if the CK19 mRNA copy number/µl lysate was less than 250 copies/µl, the result was regarded as negative (-), indicating non-metastasis; copy numbers between 250 and 5000/µl were regarded as positive (+), indicating micrometastasis; and copy numbers of 5000/µl and greater as strongly positive (++), indicating macrometastasis.

### Variables and Outcomes

The primary outcomes for this study were overall survival (OS), disease-free survival (DFS), cumulative local recurrences, and cumulative distant recurrences. In addition, the following information was collected: patient age, body mass index (BMI), tobacco smoke habit, family history of breast and ovarian cancer, previous use of estrogens, post-menopausal status, definitive type of breast surgery, definitive type of axilla surgery, definitive histological results, non-surgical treatments (e.g., neo-adjuvant or adjuvant chemotherapy), the presence of comedo-like necrosis, multifocality/multicentricity, extensive intraductal component, peritumoral vascular invasion, peritumoral inflammation, breast cancer molecular subtype, tumor grading, lymph node characteristics (e.g., presence of isolated tumor cells (ITCs), micrometastasis, extracapsular lymph node invasion, or lymph node bunching), tumor size, nodal status, and TNM stage.

The tumor stage was defined according to the VII edition of the TNM classification (AJCC/UICC) (23). Tumor histology was interpreted and classified according to the World Health Organization (24). Furthermore, Elston and Ellis’s recommendations were used to evaluate the tumor grade (25). According to Rosen and Oberman’s criteria, the peritumoral vascular invasion was considered, and the molecular subtype of breast cancer was evaluated as previously described (25, 26). In addition, the expression and quantification of ER, PR, Her-2/Neu, and the proliferative tumor fraction (Mib1/Ki67) were evaluated as previously described (26). In addition, the lymph node extracapsular invasion was defined as the extracapsular growth of tumor cells, invasion of perinodal fat, or extranodal location of tumor cells (26).

### Statistical Methods

Statistical analysis was performed using R (version 3.6.2 – http://www.R-project.org/). The normal distribution of considered numeric variables was evaluated through the Kolmogorov-Smirnov test. Numeric variables were described with the mean (± standard deviation) or median and interquartile range (IQR), while categorical variables were described as percentages and absolute values. Moreover, the following statistical tests were applied when appropriate: Wilcoxon test, t-test, Kruskall-Wallis test, and one-way ANOVA for continuous variables, Fisher exact test, or chi-square test for categorical variables. The Kaplan-Meier analysis was used to analyze overall survival, disease-free survival, and cumulative local or distant recurrences. The differences between different groups were tested using the Log-rank test. Furthermore, the univariate and multivariate Cox proportional hazards regression analysis was performed considering as response variables OS and DFS.

## Results

We included in this study 2412 patients with invasive breast cancer classified as TNM stage I and II and operated on during the considered period. A definitive histological examination of SLNB was performed in 727 cases (group A), intra-operative FS in 697 patients (group B), and OSNA in 988 cases (group C).

Mean patient age resulted in 60.24 years ( ± 12.1), mean BMI was 25.23 kg/m2 ( ± 4.77), and 79.35% of women were in their post-menopausal period. The prevalence of familial cancer history and previous use of estrogens were respectively 30.29% and 35.28%. In most cases, definitive breast surgery was conservative in most cases (62.94%), while mastectomy was definitively performed in 37.06% of cases. Adjuvant hormonal therapy was administered in 84.82% of women, adjuvant radiotherapy in 62.16%, and adjuvant chemotherapy in 31.84% (759/2384).

ITCs were found in 2.32% of cases, micrometastasis in 9.12%, and macrometastases in 13.64%. The extracapsular lymph node invasion was found in 0.5% of cases, and non-axillary loco-regional lymph node metastases were found in 1.37% of cases. Definitive CALND was performed in 22.18% of patients. Among 535 CALND, 312 were performed after detecting macrometastases, 173 micrometastases, 9 ITC, and 41 cases because of sentinel node detection failure. In addition, CALND was not performed in 47 patients with ITCs and 47 with micrometastases.

The most frequent histotype was invasive carcinoma non-special type (previously named invasive ductal carcinoma, 79.1%), followed by invasive lobular carcinoma (12.73%), other special types of invasive carcinoma (3.98%), and the combined ductal and lobular invasive carcinoma (4.19%). The most common molecular subtype was luminal A (50.08%), followed by luminal B (27.57%), basal-like (7.59%), luminal Her (5.85%), and Her-enriched (3.23%). In 5.68%, the molecular subtype was not specified. Tumor grading G2 accounted for 59.37% of cases. The majority of tumors were classified as T1 (85.66%), N0 (77.24%), and TNM stage I (75.17%).

All patients with definitive histological examination had two surgical interventions, while FS and OSNA were performed in the same surgical session as the primary breast tumor. In addition, the surgical operation was significantly longer in cases assessed intra-operatively by FS than by OSNA (70.1 minutes ±10.5 vs. 42.1 minutes ±5.1; p <0.05).

In **Table 1**, we report the different characteristics of the three studied groups. The definitive histological examination group had a significantly higher prevalence of BCS than FS or OSNA ones. In addition, FS was associated with a lower prevalence of CALND than the definitive histological examination or OSNA (**Table 1**). The prevalence of Mib-1>20% was significantly higher in OSNA than FS and definitive histological examination. **Table 2** shows the differences in terms of tumor characteristics between the definitive histological examination and OSNA or FS groups. The prevalence of luminal A subtype was significantly higher in FS than in definitive histological examination and OSNA (**Table 2**). In addition, the prevalence of positive nodes was significantly higher in the OSNA group than in the other two groups (**Table 2**). Concurrently, the OSNA group had a significantly higher prevalence of N1 tumors (**Table 3**) and a higher prevalence of both macro- and micro-metastases (**Table 2**) than the other two groups.

**Table 1:** Description of the population subdivided in the three considered groups (definitive histology, intraoperative frozen section, and OSNA).

	Definitive histology (727)	Intraoperative frozen section (697)	Intraoperative OSNA (988)	p
Age (years)	60.7 ( ± 12.0)	59.4 ( ± 11.4)	60.5 ( ± 12.6)	1
BMI (kg/m²)	25.6 ( ± 4.7)	25.1 ( ± 4.7)	24.9 ( ± 4.9)	1,2
Tobacco smoke	7.8% (53/676)	8.1% (52/644)	16.8% (90/537)	2,3
Familial cancer history	29.2% (42/144)	35.0% (82/234)	29.1% (237/814)	NS
Previous use of estrogens	33.6% (36/107)	31.1% (37/119)	39.3% (66/168)	NS
Post-menopausal status	81.4% (592/727)	80.1% (558/697)	77.3% (764/988)	2
Definitive breast surgical intervention				
BCS	74.7% (543/727)	63.1% (440/697)	54.1% (535/988)	1,2,3
Mastectomy	25.3% (184/727)	36.9% (257/697)	45.9% (453/988)	1,2,3
Definitive CALND	23.2% (169/727)	19.2% (134/697)	23.5% (232/988)	3
Non-surgical treatments				
Adjuvant radiotherapy	69.2% (496/717)	66.3% (460/694)	54.1% (526/973)	2,3
Adjuvant chemotherapy	34.3% (246/717)	29.1% (202/694)	32.0% (311/973)	1
Adjuvant hormonal therapy	83.3% (597/717)	85.0% (590/694)	85.8% (836/974)	NS

**Differences statistically significant (p < 0.05) between**, (1) definitive histology and intraoperative frozen section; (2) definitive histology and OSNA; (3) intraoperative frozen section and OSNA.

BMI, body mass index; BCS, breast conservative surgery; CALND, complete axillary lymph node dissection.

**Table 2:** Tumor characteristics considering the three groups (definitive histology, intraoperative frozen section, and OSNA).

	Definitive histology (727)	Intraoperative frozen section (697)	Intraoperative OSNA (988)	p
**Histological type**				
Invasive carcinoma non-special type	76.6% (557/727)	80.6% (562/697)	79.9% (789/988)	NS
Lobular invasive carcinoma	14.4% (105/727)	12.1% (84/697)	11.9% (118/988)	NS
Ductal and lobular invasive carcinoma	3.9% (28/727)	3.9% (27/697)	4.7% (46/988)	NS
Other invasive carcinoma	5.1% (37/727)	3.4% (24/697)	3.5% (35/988)	NS
**Tumor characteristics**				
Mib-1>20%	28.3% (196/693)	24.0% (151/628)	33.6% (320/953)	2,3
Comedo-like necrosis	4.1% (30/727)	9.9% (69/697)	8.3% (82/988)	1,2
Multifocality/multicentricity	16.6% (121/727)	14.2% (99/697)	17.1% (169/988)	NS
EIC	19.8% (144/727)	28.3% (197/697)	16.8% (166/988)	1,3
PVI	2.3% (17/727)	15.4% (107/697)	25.6% (253/988)	1,2,3
Peri-tumoral inflammation	3.3% (24/727)	0.6% (4/697)	0.5% (5/988)	1,2
**Molecular subtype**				
Luminal A	51.3% (356/694)	59.5% (377/634)	50.2% (475/947)	1,3
Luminal B	30.5% (212/694)	23.2% (147/634)	32.3% (306/947)	1,3
Luminal Her	6.1% (42/694)	6.0% (38/634)	6.4% (61/947)	NS
Her enriched	2.9% (20/694)	3.2% (20/634)	4.0% (38/947)	NS
Basal-like	9.2% (64/694)	8.2% (52/634)	7.1% (67/947)	NS
**Lymph node characteristics**				
Sentinel nodes removed >2	17.83% (64/359)	9.61% (67/697)	8.04% (78/970)	1,2
Ppositive sentinel nodes	22.0% (160/727)	17.1% (119/697)	27.3% (270/988)	1,2,3
ITC	5.6% (41/727)	2.2% (15/697)	—	1
Micrometastasis	6.5% (47/727)	5.2% (36/697)	13.9% (137/988)	2,3
Macrometastasis	15.5% (113/727)	11.9% (83/697)	13.5% (133/988)	1
Extracapsular lymph node invasion	0.6% (4/727)	0.3% (2/697)	0.6% (6/988)	NS
Non axilla locoregional lymph node metastasis	2.2% (16/727)	2.4% (17/697)	0.0% (0/988)	2,3

**Differences statistically significant (p < 0.05) between**, (1) definitive histology and intraoperative frozen section; (2) definitive histology and OSNA; (3) intraoperative frozen section and OSNA.

EIC, extensive intraductal component; PVI, peritumoral vascular invasion; ITC, isolated tumor cells.

**Table 3:** TNM stage and tumor grading among the three considered groups (definitive histology, intraoperative frozen section, and OSNA).

	Definitive histology (727)	Intraoperative frozen section (697)	Intraoperative OSNA (988)	p
Tumor local extension				
T1	83.9% (610/727)	89.2% (622/697)	84.4% (834/988)	1,3
T2	16.1% (117/727)	10.8% (75/697)	15.3% (151/988)	1,3
T3	0.0% (0/727)	0.0% (0/697)	0.3% (3/988)	NS
Nodal status				
N0	78.0% (567/727)	82.9% (578/697)	72.7% (718/988)	1,2,3
N1	21.6% (157/727)	17.1% (119/697)	27.1% (268/988)	1,2,3
N2	0.4% (3/727)	0.0% (0/697)	0.2% (2/988)	NS
TNM stage				
I	72.1% (524/727)	79.9% (557/697)	74.1% (732/988)	1,3
II	27.9% (203/727)	20.1% (140/697)	25.9% (256/988)	1,3
Tumor grading				
G1	5.8% (42/727)	28.4% (198/697)	23.9% (236/988)	1,2,3
G2	69.6% (506/727)	53.7% (374/697)	55.9% (552/988)	1,2
G3	24.6% (179/727)	17.9% (125/697)	20.2% (200/988)	1,2

**Differences statistically significant (p < 0.05) between**: (1) definitive histology and intraoperative frozen section; (2) definitive histology and OSNA; (3) intraoperative frozen section and OSNA. TNM, Tumor-Node-Metastasis.

**Figure 1:**
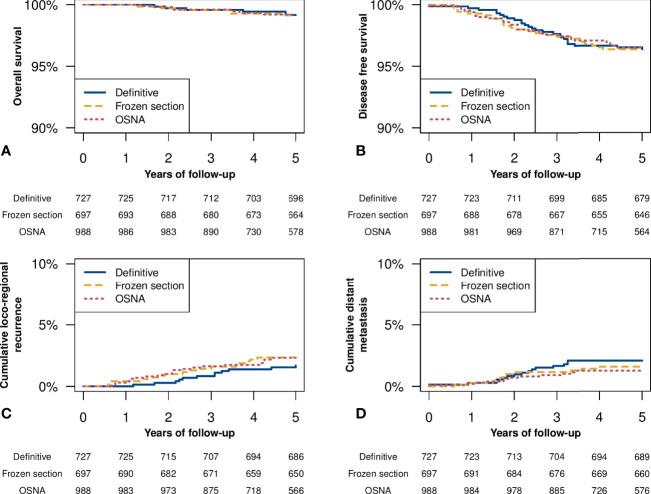
Kaplan Meier analysis. **(A)** Overall survival among the studied groups (log-rank test p-value=0.806). **(B)** Disease free survival among the studied groups (log-rank test p-value=0.295). **(C)** Cumulative loco-regional recurrence among the studied groups (log-rank test p-value=0.152). **(D)** Cumulative distant metastases among the studied groups (log-rank test p-value=0.589).

**Figure 1** shows the Kaplan-Meier analysis, and no significant differences have been observed in OS, DSF, cumulative local or distant metastases (**Figures 1A–D**). At 5 years follow-up the OS in definitive histological examination group, FS, and OSNA was respectively 99.16% (95% C.I. 98.49-99.83%), 99.12% (95% C.I. 98.43-99.82%), and 99.20% (95% C.I. 98.61-99.80%) while the DFS was respectively 96.38% (95% C.I. 95.02-97.75%), 96.37% (95% C.I. 94.98-97.78%), and 96.51% (95% C.I. 95.32-97.72%). The mortality rates in the definitive histological examination group, FS, and OSNA were respectively 1.681 deaths/1000 patients/year, 1.757 deaths/1000 patients/year, and 1.600 deaths/1000 patients/year. The local recurrence incidence rates in the definitive histology group, FS, and OSNA were respectively 3.383 cases/1000 patients/year, 4.740 cases/1000 patients/year, and 4.853 cases/1000 patients/year. The distant metastases recurrence incidence rates in the definitive histological examination group, FS, and OSNA were respectively 4.236 cases/1000 patients/year, 3.237 cases/1000 patients/year, and 2.753 cases/1000 patients/year. **Table 4** also shows univariate and multivariate cox analysis, and no significant differences have been found in OS and DFS among the three studied groups. The analysis in **Table 4** was stratified for N0, and no significant differences were found. DFS was assessed separately for macrometastases. In the univariate analysis and multivariate analysis, no significant differences were observed. The multivariate adjustment for DFS in the sub-group of macrometastases resulted for intraoperative FS of HR 2.44 (95% C.I. 0.44 - 13.38) (p=0.305) in reference to definitive histology and for Intraoperative OSNA of HR 1.05 (95% C.I. 0.18 - 6.1) (p=0.957). The multivariate adjustments were performed according to the most predictive factors and the possible confounders found in the univariate analysis. No other stratifications were performed due to the limited number of events.

**Table 4:** Univariate and multivariate Cox analysis.

OS (All nodal status)	HR (95% CI)	p	HR (95% CI) (*)	p
Definitive histology	Reference	1.000	Reference	1.000
Intraoperative FS	1.05 (0.34 - 3.25)	0.937	2.53 (0.75 - 8.55)	0.135
Intraoperative OSNA	0.97 (0.33 - 2.90)	0.962	1.54 (0.44 - 5.35)	0.498
**OS (Only N0)**	**HR (95% CI)**	**p**	HR (95% CI) (**)	**p**
Definitive histology	Reference	1.000	Reference	1.000
Intraoperative FS	0.82 (0.22 - 3.05)	0.767	1.38 (0.34 - 5.65)	0.651
Intraoperative OSNA	1.25 (0.4 - 3.96)	0.701	1.91 (0.51 - 7.06)	0.335
**DFS (All nodal status)**	**HR (95% CI)**	**p**	HR (95% CI) (*)	**p**
Definitive histology	Reference	1.000	Reference	1.000
Intraoperative FS	1.01 (0.58 - 1.74)	0.982	0.99 (0.53 - 1.86)	0.973
Intraoperative OSNA	0.96 (0.57 - 1.62)	0.888	0.84 (0.47 - 1.52)	0.573
**DFS (Only N0)**	**HR (95% CI)**	**p**	HR (95% CI) (**)	**p**
Definitive histology	Reference	1.000	Reference	1.000
Intraoperative FS	1.08 (0.57 - 2.06)	0.807	1.18 (0.56 - 2.49)	0.658
Intraoperative OSNA	1.02 (0.55 - 1.91)	0.943	1.14 (0.56 - 2.29)	0.717
**DFS (Nodal macrometastases)**	**HR (95% CI)**	**p**	HR (95% CI) (**)	**p**
Definitive histology	Reference	1.000	Reference	1.000
Intraoperative FS	0.98 (0.31 - 3.08)	0.971	2.44 (0.44 - 13.38)	0.305
Intraoperative OSNA	0.51 (0.15 - 1.74)	0.281	1.05 (0.18 - 6.1)	0.957

(*) Multivariate Cox analysis adjusted for woman age, histological type, molecular subtype, nodal status, TNM stage, tumor grading, Mib-1>20%, comedo-like necrosis, multifocality/multicentricity, EIC, PVI, type of breast surgery, type of axilla surgery.

(**) Multivariate Cox analysis adjusted for woman age, histological type, molecular subtype, TNM stage, tumor grading, Mib-1>20%, comedo-like necrosis, multifocality/multicentricity, EIC, PVI, type of breast surgery, type of axilla surgery.

## Discussion

Surgical procedure length was significantly shortened by the intraoperative OSNA technique. In addition, despite the long follow-up considered, no significant differences have been observed among the three groups (intraoperative OSNA or FS and definitive histology) regarding OS, DSF, cumulative local or distant metastases, apart from a non-significant increased risk of local recurrences related to FS and OSNA method.

Previous studies demonstrated a peak of local recurrences after SLNB between the third and the sixth year of follow-up, which are mostly included in our study (11). Only a limited number of studies performed a survival analysis considering OSNA, and none compared in the same population in all three groups we considered based on the SLNB evaluation technique (10, 27, 28). Recently, Shimazu and coworkers found a significantly improved DFS in N0 status detected by OSNA, if compared with traditional histology, suggesting the better staging efficiency of the OSNA method (28). Our data did not confirm this advantage. Instead, we showed a non-significant increased recurrence risk in the case of intraoperative OSNA, in comparison with definitive histology, which may be explained by the higher incidence of unfavorable prognostic factors found in the group of patients who underwent OSNA evaluation of their SLNB. Indeed, the OSNA method resulted significantly associated with tumor features, which are usually expressions of a more aggressive biological behavior of the disease, such as tumor multifocality/multicentricity, extensive intraductal component, peritumoral vascular invasion, comedo-like necrosis, and Mib-1>20% (29). And in our opinion, this fact simply reflects the progressive extension of SLNB indications.

In the literature, the OSNA system allowed a more efficient detection of micrometastasis, consequently decreasing the number of false-negative histological examinations resulting from the small size of micrometastases, which may not be included in any microscopical section (28, 30, 31). Also, in our experience, a significant increase in the prevalence of micrometastasis was found compared to FS and definitive histology. In addition, the prevalence of macrometastases was similar to the definitive histology, while FS had a significantly lower prevalence of macrometastases than definitive histology. This last finding could be due to a significantly higher detection rate of definitive histology than FS or simply to a better selection of subjects undergoing FS than definitive histology. Along with the increased number of detected micrometastases compared to FS and definitive histology, the OSNA technique also increased the number of diagnosed macrometastases than FS, correlating with a higher prevalence of node-positive disease and consequently a higher prevalence of secondary CALNDs. However, Hintzen and coworkers recently demonstrated that the increased rate of CALND after OSNA could be limited by broader adoption of the criteria that emerged from the Z0011 and AMAROS trials for axilla treatment (32–34). In particular, they found that the use of the OSNA method, in association with these emerging criteria for axilla treatment, does not lead to more CALNDs, axilla radiotherapy, or adjuvant systemic therapies (34).

As expected, the OSNA technique resulted in an evident improvement in breast surgery in our center. In particular, in accordance with the literature (4), it succeeded in significantly reducing the surgical time from a mean operation length of 70.1 ( ± 10.5) minutes in the case of FS to a mean operation length of 42.1 ( ± 5.1) minutes using OSNA. However, this result required accurate compliance with some technical premises, such as the strong limitation of the number of excised nodes. Consequently, both OSNA and FS correlated with a smaller number of excised sentinel nodes than definitive histological examination, resulting in nearly one single node. Furthermore, recently Saruta and coworkers found that in Japan, the adoption of the OSNA technique, in addition to reducing the burden on the patient (limiting the number of surgeries and the duration of surgical procedures), also reduced the breast cancer healthcare costs per patient (35).

The main limitations of this study are the retrospective and non-randomized nature of the chart review and the unavailability of detailed data about cost-effectiveness and side effects. Among the strengths of this study, we can emphasize the broad cohort and the remarkable follow-up data. In addition, another essential strength is the uniform management due to regular multidisciplinary meetings in a single-center experience.

Our findings add further proof to the body of evidence supporting the wider adoption of this innovative technology that enables a safe reduction in patient surgical burden and healthcare costs. The reduction in costs also comprises a lower workload for the pathologist than intraoperative FS and definitive histology.

In conclusion, no difference was found in OS and DFS when comparing OSNA, FS, and definitive histology. At the same time, the OSNA system was advantageous in reducing single-session surgical operating time and the pathologist workload.

## Data Availability Statement

The datasets presented in this article are not readily available because the data that support the findings of this study are available, but restrictions apply to the availability of these data, which was used under license for the current study, and so are not publicly available. Data are however available from the authors upon reasonable request and with permission of the Internal Review Board. Requests to access the datasets should be directed to serena.bertozzi@asufc.sanita.fvg.it.

## Ethics Statement

The studies involving human participants were reviewed and approved by Internal Review Board of the Department of Medical Area (University of Udine). Written informed consent for participation was not required for this study in accordance with the national legislation and the institutional requirements.

## Author Contributions

Substantial contributions to conception and design or acquisition of data or to analysis and interpretation of data: SB, AL, MB, LS, DA, AP, MA, MO, LM, and CC. Drafting the article or revising it critically for important intellectual content: SB, AL, MB, LS, DA, AP, MA, MO, LM, and CC. All authors have read and approved the final manuscript.

## Conflict of Interest

The authors declare that the research was conducted in the absence of any commercial or financial relationships that could be construed as a potential conflict of interest.

## Publisher’s Note

All claims expressed in this article are solely those of the authors and do not necessarily represent those of their affiliated organizations, or those of the publisher, the editors and the reviewers. Any product that may be evaluated in this article, or claim that may be made by its manufacturer, is not guaranteed or endorsed by the publisher.

## Abbreviations

AJCC/UICC, American Joint Committee on Cancer/Union for International Cancer Control; BMI, body mass index; CALND, complete axillary lymph node dissection; CI, confidence interval; CK, cytokeratin; DFS, disease free survival; FS, frozen section; IQR, interquadrtile range; ITC, isolated tumor cell; mRNA, messenger ribonucleic acid; OS, overall survival; OSNA, One-step nucleic acid amplification; SLNB, sentinel lymph node biopsy; TNM, tumor, node, metastasis.
